# Hyperemic myocardial blood flow in patients with atrial fibrillation before and after catheter ablation: A dynamic stress CT perfusion study

**DOI:** 10.14814/phy2.15123

**Published:** 2021-11-21

**Authors:** Masafumi Takafuji, Kakuya Kitagawa, Satoshi Nakamura, Takanori Kokawa, Yoshihiko Kagawa, Satoshi Fujita, Tomoyuki Fukuma, Eitaro Fujii, Kaoru Dohi, Hajime Sakuma

**Affiliations:** ^1^ Department of Radiology Mie University Graduate School of Medicine Tsu Japan; ^2^ Department of Cardiology and Nephrology Mie University Graduate School of Medicine Tsu Japan

**Keywords:** atrial fibrillation, CT delayed enhancement, dual‐source CT, dynamic CT perfusion, myocardial blood flow

## Abstract

**Background:**

Atrial fibrillation (AF) patients without coronary artery stenosis often show clinical evidence of ischemia. However myocardial perfusion in AF patients has been poorly studied. The purposes of this study were to investigate altered hyperemic myocardial blood flow (MBF) in patients with AF compared with risk‐matched controls in sinus rhythm (SR), and to evaluate hyperemic MBF before and after catheter ablation using dynamic CT perfusion.

**Methods:**

Hyperemic MBF was quantified in 87 patients with AF (44 paroxysmal, 43 persistent) scheduled for catheter ablation using dynamic CT perfusion, and compared with hyperemic MBF in 87 risk‐matched controls in SR. Follow‐up CT after ablation was performed in 49 AF patients.

**Results:**

Prior to ablation, hyperemic MBF of patients in AF during the CT (1.29 ± 0.34 ml/mg/min) was significantly lower than in patients in SR (1.49 ± 0.26 ml/g/min, *p* = 0.002) or matched controls (1.65 ± 0.32 ml/g/min, *p* < 0.001); no significant difference was seen between patients in SR during the CT and matched controls (vs. 1.50 ± 0.31 ml/g/min, *p* = 0.815). In patients in AF during the pre‐ablation CT (*n* = 24), hyperemic MBF significantly increased after ablation from 1.30 ± 0.35 to 1.53 ± 0.17 ml/g/min (*p* = 0.004); whereas in patients in SR during the pre‐ablation CT (*n* = 25), hyperemic MBF did not change significantly after ablation (from 1.46 ± 0.26 to 1.49 ± 0.27 ml/g/min, *p* = 0.499).

**Conclusion:**

In the current study using stress perfusion CT, hyperemic MBF in patients with AF during pre‐ablation CT was significantly lower than that in risk‐matched controls, and improved significantly after restoration of SR by catheter ablation, indicating that MBF abnormalities in AF patients are caused primarily by AF itself.

## 1 INTRODUCTION

Atrial fibrillation (AF) is the most common persistent arrhythmia in the elderly and is on the rise worldwide (Chugh et al., 2014). Coronary artery disease is a common comorbidity of AF, occurring in 17%–46.5% of patients with AF (Michniewicz et al., 2018), but, it is also known that AF patients without coronary artery disease often show clinical evidence of ischemia, such as chest pain, ST depression, or troponin release at the onset of arrhythmia (Bos et al., 2011; Parwani et al., 2013). Indeed, previous studies using PET and MRI have shown a decrease in hyperemic myocardial blood flow (MBF) and myocardial perfusion reserve in patients with AF (Byrne et al., 2019; Pantlin et al., 2020; Range et al., 2007; Sugimoto et al., 2021; Wijesurendra et al., 2018). Impaired myocardial perfusion may be attributable to the arrhythmia itself or to microvascular dysfunction associated with AF (Byrne et al., 2019; Pantlin et al., 2020; Range et al., 2007; Sugimoto et al., 2021; Wijesurendra et al., 2018). Catheter ablation has been established as an effective treatment for patients with AF. However, to the best of our knowledge, only two studies have examined whether catheter ablation improves myocardial perfusion, and conflicting results have been obtained regarding the effect of catheter ablation on MBF (Range et al., 2007; Wijesurendra et al., 2018). Cardiac CT is frequently used to delineate the complex anatomy of the left atrium and pulmonary veins prior to catheter ablation and to evaluate coronary artery disease. Moreover, MBF can be quantified with the addition of CT myocardial perfusion imaging (CTP; Bamberg et al., 2014). In this study, we quantitatively measured hyperemic MBF before and after catheter ablation using CTP to assess the effect of restoring sinus rhythm (SR).

## 2 METHODS

### 2.1 Study population

We performed a sub‐analysis in a prospective, single center study to determine the predictors of recurrence of AF and long‐term prognosis. Three hundred and twenty patients with AF who underwent catheter ablation between September 2016 and December 2019 were enrolled in this study. The study was approved by the Institutional Review Board of our institution (reference number: 3084) and written informed consent for participation in the study was given by all patients. The inclusion criteria in this sub‐analysis were (1) patients who underwent cardiac CT for evaluation of left atrial anatomy and coronary artery disease prior to catheter ablation in the study period, and (2) patients who gave consent for dynamic CTP for assessment of hyperemic MBF prior to ablation. Of the 286 patients who underwent cardiac CT, 106 patients agreed to undergo dynamic CTP. Patients with any of the following were excluded from analysis: (1) previous coronary revascularization via percutaneous coronary intervention (*n* = 6) or coronary artery bypass graft (*n* = 1); (2) myocardial infarction (*n* = 1); (3) hypertrophic cardiomyopathy (*n* = 7); (4) cardiac pacemakers (*n* = 2). Therefore, the final study population consisted of 89 patients with AF. In 49 patients (23 with paroxysmal AF and 26 with persistent AF) who agreed to undergo follow‐up dynamic CTP after successful catheter ablation, post‐ablation cardiac CT was performed using the same protocol after a median interval of 3.3 months from the pre‐ablation CT (interquartile range, 2.6–5.3 months). Clinical management, including ablation strategy and medical therapy, was at the discretion of the responsible physician. Pulmonary vein isolation was the mainstay of the catheter ablation procedure and was undertaken in all patients. Radiofrequency ablation was performed in 33 patients (67%), cryoballoon ablation in 10 patients (20%), and hot balloon ablation in six patients (12%).

**FIGURE 1:**
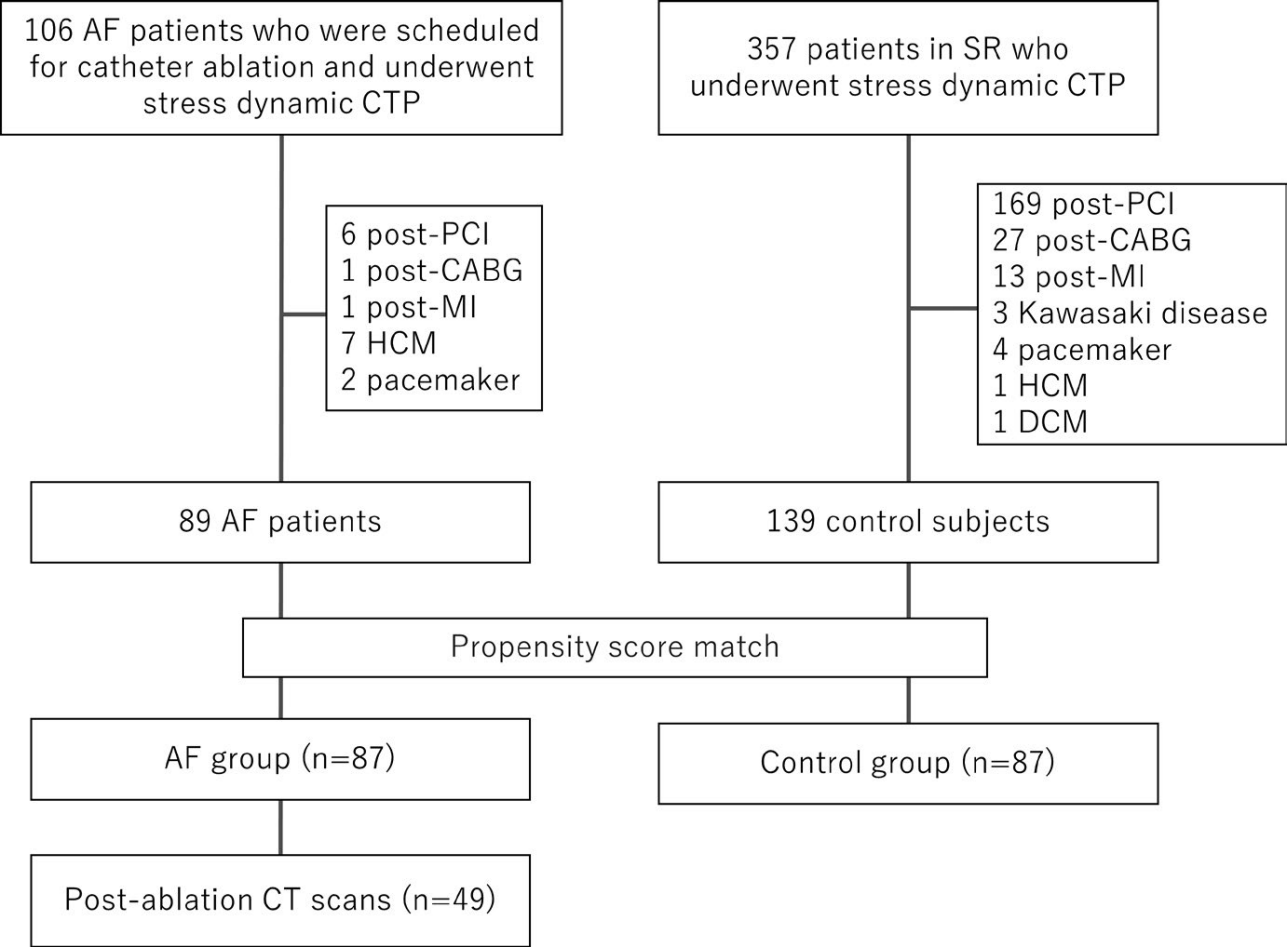
Flowchart showing the patient selection protocol for patient enrollment. AF, atrial fibrillation; CABG, coronary artery bypass graft; DCM, dilated cardiomyopathy; HCM, hypertrophic cardiomyopathy; MI, myocardial infarction; PCI, percutaneous coronary intervention; SR, sinus rhythm

We established a control group in SR by propensity risk score matching for comparison of hyperemic MBF. The mother population of the control group for matching was 357 patients who underwent stress perfusion CT to evaluate coronary artery disease in the same period (between September 2016 and December 2019). The exclusion criteria for controls in SR were the same as for patients with AF. The final number of patients eligible for matching were 139. The inclusion flowchart is shown in Figure 1.

### 2.2 CT protocol and image acquisitions

All CT examinations were performed using a third‐generation dual‐source CT (SOMATOM Force; Siemens Healthineers). The CT protocol comprised stress dynamic CTP followed by coronary CT angiography (CCTA) and CT delayed enhancement (CTDE). Dynamic CTP was performed with a bolus injection of 40 ml of iopamidol (flow rate, 5 ml/s) with an iodine concentration of 370 mgI/ml (Iopamiron 370; Bayer‐Schering Pharma) followed by 20 ml of saline (flow rate, 5 ml/s). After administering adenosine triphosphate (ATP; Adetphos‐L; Kowa) at 0.16 mg/kg/min for 3 min, dynamic datasets were acquired for 30 s in the electrocardiography (ECG)‐triggered mode, with the table moving forward and backward between the two positions (Bamberg et al., 2011). Data were acquired at end‐systole (250 ms after the R peak) with tube voltage of 70 kVp. Tube current was determined using automatic tube current modulation with the quality reference of 300 mAs/rot at 80 kVp (Takafuji, Kitagawa, Ishida, et al., 2020). Ten minutes after dynamic stress CTP, standard prospective CCTA covering from end‐systole (250 ms after the R peak) to mid‐diastole was performed at rest with injection of 0.84 ml/kg of contrast medium over 12 s. Tube voltage was 2 × 80 or 70 kVp, and tube current was determined using automatic exposure control with the quality reference of 300 mAs/rot at 120 kVp. Seven minutes after CCTA, end‐systolic CTDE images were acquired without administration of any additional contrast medium, using ECG‐triggered axial scanning at two alternating table positions (Kurita et al., 2016; Kurobe et al., 2014; Takafuji, Kitagawa, Nakamura, et al., 2020). Three image stacks were obtained during one breath‐hold. Tube voltage was 80 kVp, and tube current was determined by automatic exposure control with quality reference mAs settings at 580 mAs/80 kVp.

### 2.3 Image reconstruction and analysis

Dynamic CTP images were reconstructed in the axial plane with 3‐mm slice thickness and 2‐mm overlap. The CTP images were analyzed to estimate MBF using commercially available software (syngo.via; Siemens Healthineers). Time attenuation curves were fitted to a simplified two‐compartment model of intra‐ and extravascular space using a dedicated parametric deconvolution technique, as described previously (Mahnken et al., 2010). MBF was calculated from the maximum upslope of the fitted model curve for every voxel. The MBF values in 16 segments (i.e., the 17 segments of the American Heart Association model except for the apex) were obtained from the MBF map. Patient‐based MBF was calculated in the remote myocardium, which was defined as the average MBF of the top three segments (Takafuji, Kitagawa, Ishida, et al., 2020). Further we measured the hyperemic coronary vascular resistance (CVR) calculated as mean arterial pressure divided by hyperemic MBF (Byrne et al., 2019; Range et al., 2007).

All CCTA images were reconstructed with a medium soft convolution kernel (Bv40) using iterative reconstruction (Advanced Modeled Iterative Reconstruction level 3). The CTA images were visually evaluated by two board‐certified radiologists (S.N. and K.K., with 6 and 15 years of experience in CCTA, respectively) who were blinded to the patient's clinical information, in joint reading. Coronary segments with a reference diameter >1.5 mm were assessed for stenosis. The severity of coronary artery disease on CTA was ranked by the Coronary Artery Disease‐Reporting and Data System; CAD‐RADS): 0 (0%), 1 (1%–24%), 2 (25%–49%), 3 (50%–69%), 4A (70%–99% in one to twovessels), 4B (70%–99% in three vessels or ≥50% left main), or 5 (100%) (Cury et al., 2016).

The CTDE images were reconstructed with 1‐mm slice thickness. The three image stacks were then averaged after nonrigid registration using volume perfusion software (syngo.via; Siemens Healthineers). The CTDE images were evaluated visually for the presence of delayed enhancement.

### 2.4 Statistical analysis

The sample size was calculated with a two‐sided test, alpha probability of 0.05 and power of 0.95. The difference to detect and the approximate estimate of the standard deviation was set at 0.44 and 0.48 on the basis of previous data (Range et al., 2007). Target sample size was 64 subjects. Expecting an exclusion rate of 25%, 87 subjects were required for each group. Continuous variables are expressed as the mean ± SD, and categorical variables are expressed as proportions. Student's *t*‐test (paired when appropriate) was used to assess differences in continuous variables after examining the normality of the data. The Chi‐squared test was used to assess differences in proportion between categorical variables. The propensity score was estimated from age, sex, BMI, hypertension, dyslipidemia, diabetes mellitus, smoking, and family history of coronary artery disease both for patients with AF and for controls in SR. We selected controls in SR against patients with AF (1:1 matching) using a nearest‐neighbor matching algorithm within a caliper of 0.25 SD of the propensity score. Univariate and multivariate logistic regression were used to analyze factors associated with MBF impairment in remote myocardium, including age, sex, BMI, time from first AF diagnosis, risk factors for coronary artery disease, CHADS_2_ score, CHA_2_DS_2_‐VASc score, medications, CAD‐RADS score, and presence of delayed enhancement. The cutoff value of MBF impairment was defined as below the 5th percentile for the control subjects. All statistical analyses were performed using JMP version 14 software (SAS Institute Inc.). Values of *p* < 0.05 were considered to indicate statistical significance.

## 3 RESULTS

Propensity score matching yielded a total of 87 matched subjects (Figure 1). There were no significant differences in clinical factors or propensity scores between patients with AF and the matched control group (Table 1). In the AF group, there were 43 patients with persistent AF and 44 patients with paroxysmal AF.

**TABLE 1:** Subject characteristics after propensity score matching

Patient characteristics	Patient with AF (*n* = 87)	Controls in SR (*n* = 87)	*p* value
Sex			0.871
Male, *n* (%)	60 (69)	59 (68)	
Female, *n* (%)	27 (31)	28 (32)	
Age (years), mean ± SD	65 ± 10	66 ± 10	0.535
Body mass index (kg/m²), mean ± SD	24.5 ± 4.1	24.2 ± 4.2	0.748
Risk factor, *n* (%)			
Hypertension	50 (57)	53 (61)	0.644
Diabetes mellitus	14 (16)	15 (17)	0.839
Hyperlipidemia	32 (37)	35 (40)	0.640
Smoking	47 (54)	46 (53)	0.879
Family history of CAD	9 (10)	9 (10)	1.000
Propensity score, mean ± SD	0.43 ± 0.10	0.42 ± 0.10	0.408
Medication, *n* (%)			
ACE‐I/ARB	29 (33)	23 (26)	0.320
Beta‐blockers	34 (39)	13 (15)	<0.001
Diuretics	8 (9)	6 (7)	0.577
Calcium channel blockers	31 (36)	27 (31)	0.520
Natrium channel blocker	21 (24)	0 (0)	<0.001
Digoxin	5 (6)	0 (0)	0.023
Statin	26 (30)	26 (30)	1.000
CAD‐RADS score, *n* (%)			0.126
0	33 (38)	23 (26)	
1	22 (25)	18 (21)	
2	19 (22)	17 (20)	
3	7 (8)	17 (29)	
4A	5 (6)	9 (10)	
4B	1 (1)	3 (3)	
5	0 (0)	0(0)	
Delayed enhancement	8 (9)	5 (6)	0.387

Abbreviations: ACE‐I/ARB, angiotensin converting enzyme inhibitor/angiotensin II receptor blocker; AF, atrial fibrillation; CAD, coronary artery disease; CAD‐RADS: Coronary Artery Disease‐Reporting and Data System; SD, standard deviation; SR, sinus rhythm.

After administration of ATP, heart rate increased from 68 ± 13/min (41–110/min) to 78 ± 15/min (34–118/min) in the 87 patients with AF (*p* < 0.001), and from 67 ± 12/min (44–103/min) to 82 ± 13/min (59–125/min) in the 87 controls in SR (*p* < 0.001). At the time of the CT scan before catheter ablation, 41 patients (39 with persistent AF and two with paroxysmal AF) were in AF, whereas the remaining 46 (four with persistent AF and 42 with paroxysmal AF) were in SR. Table 2 summarize the hemodynamic data during CT scan.

**TABLE 2:** Hemodynamic data during CT scan

	Patient with AF (*n* = 87)	Controls in SR (*n* = 87)	*p* value
AF rhythm, *n* (%)	41 (47)	0 (0)	<0.001
Rest			
Systolic BP	117 ± 16	122 ± 18	0.041
Diastolic BP	67 ± 11	65 ± 12	0.219
Mean BP	84 ± 11	84 ± 12	0.874
HR	68 ± 13	67 ± 12	0.484
Stress			
Systolic BP	106 ± 15	109 ± 20	0.185
Diastolic BP	60 ± 11	57 ± 11	0.040
Mean BP	76 ± 11	74 ± 12	0.509
HR	78 ± 15	82 ± 13	0.087

Note

Values are mean ± SD unless else is indicated.

Abbreviations: AF, atrial fibrillation; BP, blood pressure; HR, heart rate; SR, sinus rhythm.

The dose–length products of dynamic CTP and the entire protocol were 252 ± 75 mGy cm and 1213 ± 400 mGy cm, respectively.

### 3.1 MBF in patients with AF

**FIGURE 2:**
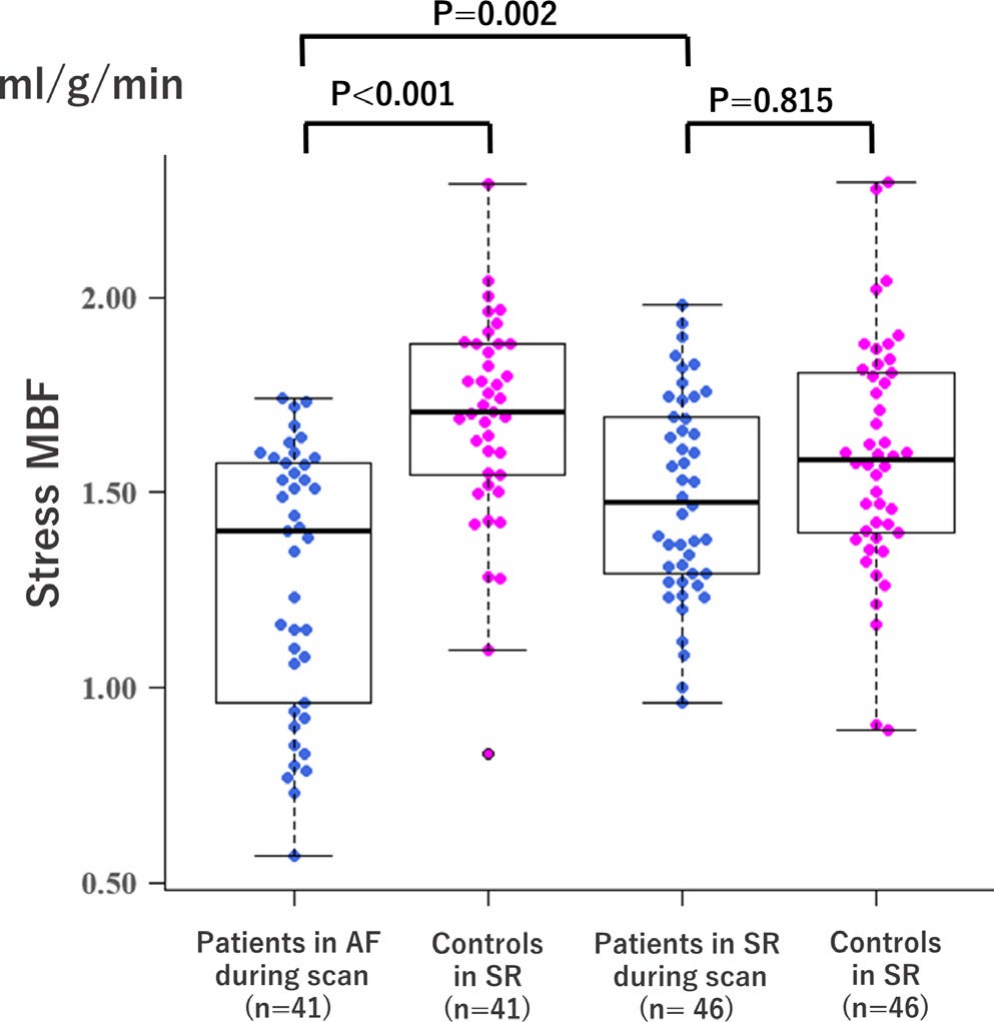
Bee swarm plots and box plots comparing hyperemic MBF in patients in AF and SR during pre‐ablation CT, and risk‐matched controls in SR. The center lines are the medians, the box limits indicate the 25th and 75th percentiles, and the whiskers extend 1.5‐times interquartile range from the 25th to 75th percentiles. MBF, myocardial blood flow; all other abbreviations as in Figure 1

Mean MBF during pre‐ablation CT of the 87 AF patients (1.39 ± 0.31 ml/g/min) was significantly lower than that of the 87 matched controls (1.57 ± 0.32 ml/g/min, *p* < 0.001). When the AF patients were divided into two groups according to the presence or absence of AF during pre‐ablation CT scan, mean MBF was significantly lower in the 41 AF patients who were in AF during pre‐ablation CT (1.29 ± 0.34 ml/mg/min) compared with the 46 AF patients who were in SR during pre‐ablation CT (1.49 ± 0.26 ml/g/min, *p* = 0.002) and the 41 controls matched with AF patients who were in AF during pre‐ablation CT (1.65 ± 0.32 ml/g/min, *p* < 0.001). No significant difference was seen between the 46 AF patients in SR during pre‐ablation CT and the 46 matched controls (vs. 1.50 ± 0.31 ml/g/min, *p* = 0.815; Figure 2).

Hyperemic CVR during pre‐ablation CT of the 87 AF patients [57.8 ± 19.5 mmHg/(ml/g/min)] was significantly higher than that of the 87 matched controls [49.4 ± 13.8 mmHg/(ml/g/min), *p* = 0.001]. Hyperemic CVR of the 41 AF patients who were in AF during pre‐ablation CT [65.8 ± 23.5 mmHg/(ml/g/min)] was significantly higher than those of the 46 AF patients who were in SR during pre‐ablation CT [50.7 ± 11.0 mmHg/(ml/g/min), *p* < 0.001] and the 41 controls matched with AF patients who were in AF during pre‐ablation CT [47.3 ± 11.9 mmHg/(ml/g/min), *p* < 0.001]. No significant difference was seen between the 46 AF patients in SR during pre‐ablation CT and the 46 matched controls [vs. 51.3 ± 15.2 mmHg/(ml/g/min), *p* = 0.853].

### 3.2 Factors associated with impaired hyperemic MBF in patients with AF

MBF impairment (<0.99 ml/g/min; fifth percentile for the control subjects) was observed in 12 (14%) of the 87 AF patients. Among the putative factors involved, AF during pre‐ablation CT (odds ratio [OR]: 16.50; 95% confidence interval [CI], 2.02–134.55; *p* = 0.009), time from first AF diagnosis >16 months (OR: 6.36; 95% CI, 1.30–31.06; *p* = 0.022), and presence of delayed enhancement (OR: 8.88; 95% CI, 1.85–42.52; *p* = 0.006) showed significant association with MBF impairment in univariate analysis. CAD‐RADS score was not significantly associated with MBF impairment in univariate analysis. In multivariate analysis, AF during pre‐ablation CT (OR: 10.79; 95% CI, 1.23–94.65; *p* = 0.032) and presence of delayed enhancement (OR: 7.49; 95% CI, 1.20–46.76; *p* = 0.031) was significant predictors of MBF impairment (Table 3).

**TABLE 3:** Factors associated with MBF impairment in AF patients

	Univariate analysis		Multivariate analysis	
	Odd ratio (95% CI)	*p* value	Odd ratio (95% CI)	*p* value
Male	5.84 (0.71–47.74)	0.100		
Age > 75 years	0.76 (0.09–6.70)	0.806		
Body mass index > 25 kg/m²	3.00 (0.83–10.86)	0.094		
Hypertension	1.04 (0.30–3.58)	0.948		
Diabetes mellitus	1.94 (0.45–8.31)	0.372		
Dyslipidemia	0.53 (0.13–2.12)	0.368		
Family history of CAD	3.83 (0.81–18.06)	0.089		
Smoking	2.92 (0.73–11.64)	0.129		
CHADS2 score > 1^a^	1.20 (0.35–4.14)	0.774		
CHA2DS2‐VASc score > 2^a^	1.20 (0.35–4.14)	0.774		
Medication				
ACE‐I/ARB	0.63 (0.16–2.52)	0.512		
Beta‐blockers	1.68 (0.49–5.71)	0.407		
Calcium channel blockers	0.89 (0.24–3.23)	0.858		
Natrium channel blocker	0.25 (0.03–2.06)	0.198		
Digoxin	1.61 (0.16–15.80)	0.681		
Statin	0.75 (0.19–3.04)	0.691		
AF during pre‐ablation CT	16.50 (2.02–134.55)	0.009	10.79 (1.23–94.65)	0.032
Time from first AF diagnosis > 16 months^a^	6.36 (1.30–31.06)	0.022	4.46 (0.76–26.14)	0.097
CAD‐RADS score > 2	0.48 (0.06–4.05)	0.498		
Delayed enhancement	8.88 (1.85–42.52)	0.006	7.49 (1.20–46.76)	0.031

Abbreviations: ACE‐I/ARB, angiotensin converting enzyme inhibitor/angiotensin II receptor blocker; AF, atrial fibrillation; CAD, coronary artery disease; CAD‐RADS, Coronary Artery Disease‐Reporting and Data System; CI, confidence interval.

^a^ Median of AF patient.

### 3.3 Effect of catheter ablation

Paired pre‐ and post‐ablation hyperemic MBF results were available in 49 patients; of these, 24/49 (50%) and 2/49 (4%) were in AF during the pre‐ and post‐ablation CT scans, respectively.

**FIGURE 3:**
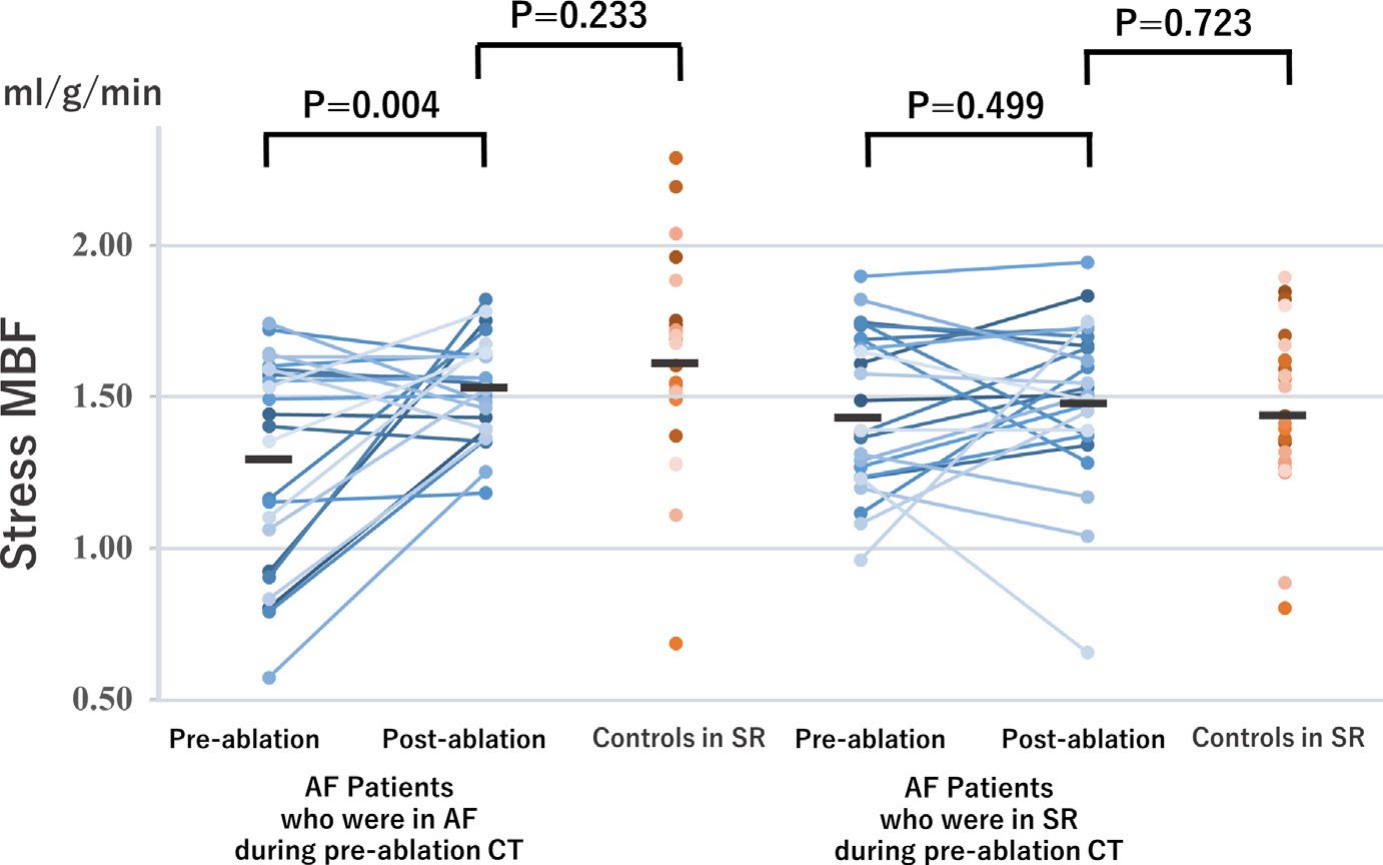
Paired pre‐ and post‐catheter ablation hyperemic MBF in patients in AF (*n* = 24) and SR (*n* = 25) during pre‐ablation CT, and propensity score‐matched controls in SR (*n* = 49). The bars indicate mean values. Abbreviations are the same as in Figures 1 and 2

**FIGURE 4:**
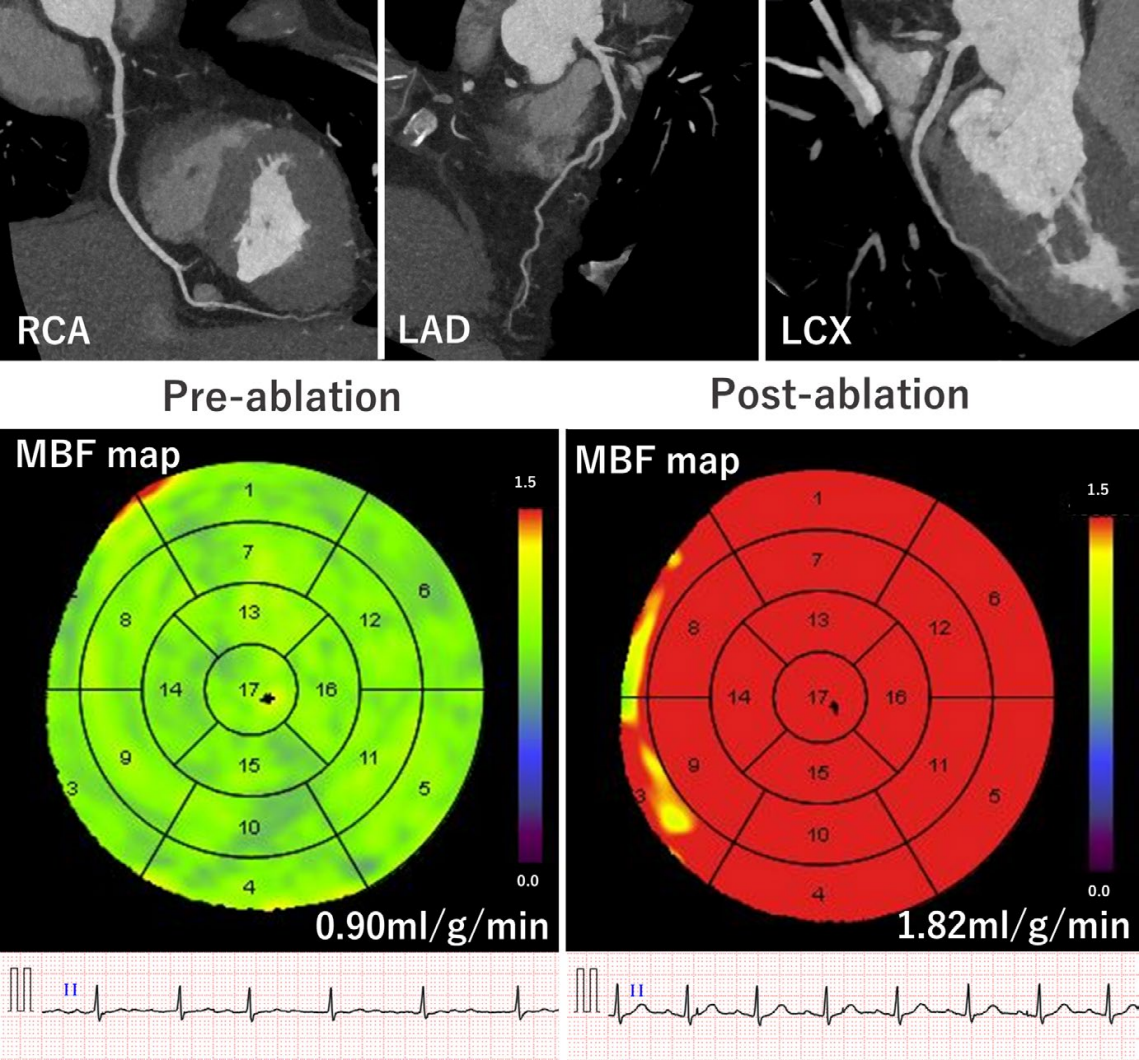
Seventy‐three‐year‐old man with persistent AF who underwent catheter ablation. Heart rhythm was AF during pre‐ablation CT and SR after ablation. Global impairment of hyperemic MBF observed before ablation despite disease‐free coronary arteries (CAD‐RADS score: 0) improved after restoration of sinus rhythm by catheter ablation. Abbreviations are the same as in Figure 3

Hyperemic MBF significantly increased after ablation from 1.30 ± 0.35 to 1.53 ± 0.17 ml/g/min (*p* = 0.004) in the 24 patients who were in AF during pre‐ablation CT, whereas no significant changes were observed in the 25 patients who were in SR during pre‐ablation CT (from 1.46 ± 0.26 to 1.49 ± 0.27 ml/g/min; *p* = 0.499). Hyperemic MBF on post‐ablation CT showed no significant difference when compared with the matched controls (*p* = 0.233 and *p* = 0.723, respectively, for patients in AF and SR during pre‐ablation CT; Figure 3). A representative case is presented in Figure 4.

Hyperemic CVR significantly decreased after ablation from 64.4 ± 22.4 to 46.7 ± 8.3 mmHg/(ml/g/min) (*p* = 0.001) in the 24 patients who were in AF during pre‐ablation CT, whereas no significant changes were observed in the 25 patients who were in SR during pre‐ablation CT [from 52.9 ± 11.1 to 50.6 ± 12.1 mmHg/(ml/g/min); *p* = 0.478]. Hyperemic MBF on post‐ablation CT showed no significant difference when compared with the matched controls (*p* = 0.908 and *p* = 0.726, respectively, for patients in AF and SR during pre‐ablation CT).

## 4 DISCUSSION

In this study, we compared hyperemic MBF during pre‐ablation CT between patients with AF and matched controls in SR. Hyperemic MBF was significantly lower in patients who were in AF than in the control group, whereas no significant difference was seen between patients in SR and matched controls during pre‐ablation CT. The MBF impairment in patients in AF during pre‐ablation CT improved to the level of the matched controls after restoration of SR with catheter ablation. To our knowledge, this is the largest study to assess myocardial perfusion in patients with AF and the first to use dynamic CTP for this purpose.

There are only two studies that evaluated MBF before and after catheter ablation in humans using PET (Range et al., 2007) and MRI (Wijesurendra et al., 2018). Range et al. (2007) reported that ^15^O‐water PET‐derived hyperemic MBF in male patients with idiopathic persistent AF was diminished by approximately 40% compared with risk‐matched controls. Hyperemic MBF improved in 10 patients who underwent follow‐up testing for 4 months after the restoration of stable SR, but remained significantly reduced when compared with matched controls. From these observations, they concluded that MBF abnormalities in AF patients are caused primarily by heart rate irregularity and are maintained by additional mechanisms such as increased sympathetic activity and longer recovery periods. Wijesurendra et al. (2018) reported that the reduction in stress MBF in patients with AF is not a direct effect of the arrhythmia itself but rather an indication of underlying coronary endothelial dysfunction, for the reason that they did not observe significant improvement of MRI‐derived hyperemic MBF after ablation in 22 cases of paroxysmal and 11 cases of persistent AF. In our present study, impaired hyperemic MBF in AF patients, particularly in those who were in AF during pre‐ablation CT, demonstrated a significant improvement to the same level of risk‐matched controls after catheter ablation, indicating that MBF abnormalities in AF patients are caused primarily by AF itself, and are reversible.

In the AF conditions, the irregularity of ventricular cycle lengths in AF proved disadvantageous for hemodynamics (Clark et al., 1997; Kochiadakis et al., 2002) and cardiac function. Furthermore, extravascular compressive forces are an important determinant of coronary blood flow when maximal coronary vasodilation is present (Laughlin et al., 2012). Hence, increased ventricular rate during AF may increase the average extravascular compression of the intramural coronary microvasculature thereby limiting hyperemic MBF. Indeed, Range et al. (2007) and Byrne et al. (2019) observed increased stress CVR in the AF group compared with patients without AF. In the current study, patients with AF were more often on beta‐blockade and sodium channel blockade in order to control ventricular rate. Although average heart rate was similar between AF and controls, these drugs involves / produce slow contraction and relaxation and prolong action potential duration, thereby prolonging systole and reducing diastolic perfusion time. The significant decrease in hyperemic CVR after ablation in our study may indicate that extravascular compressive force was increased due to prolonged systolic duration before ablation.

**FIGURE 5:**
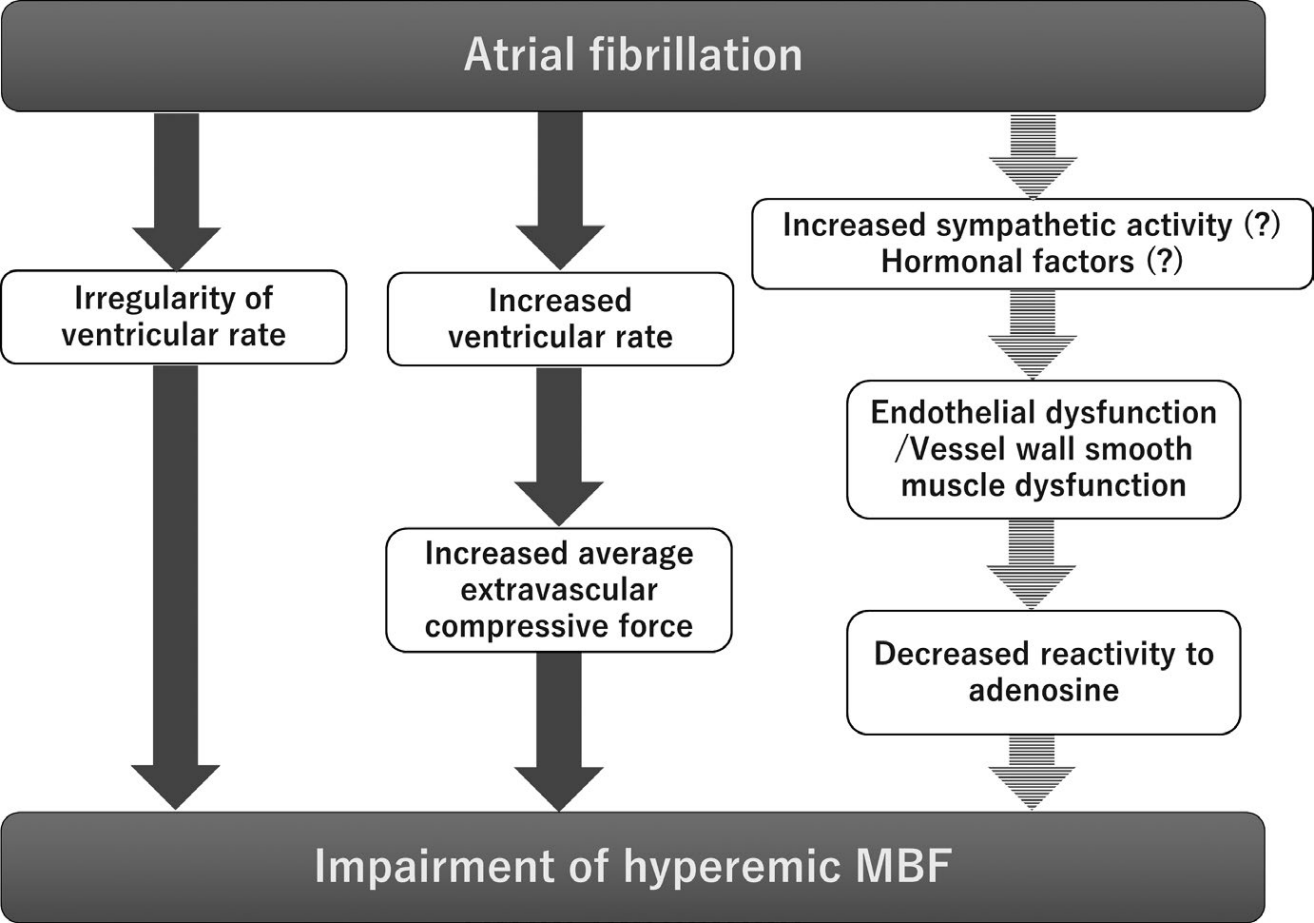
Concept map of pathophysiology of MBF impairment in patients with AF. AF, atrial fibrillation; MBF, myocardial blood flow

There is some evidence that endothelial dysfunction and vessel wall smooth muscle dysfunction occur in AF (Range et al., 2007; Wijesurendra et al., 2018). The mechanism of microvascular dysfunction in AF has not been elucidated in detail, but it has been suggested that sympathetic hyperactivity and hormonal factors are involved (Range et al., 2007). If microvascular dysfunction is caused by AF, it would be expected that the vascular reactivity to adenosine would be reduced, resulting in a reduction of the hyperemic MBF. In our study, however, despite the use of ATP, a precursor of adenosine, no persistent impairment of MBF was observed after ablation, and potential involvement of microvascular dysfunction was not demonstrated. A concept map showing the pathophysiology of MBF impairment in patients with AF was shown in Figure 5.

Previous studies have reported delayed LV enhancement by MRI in 6%–13% of patients with AF despite the absence of prior myocardial infarction (Neilan et al., 2013; Suksaranjit et al., 2015), and reported delayed LV enhancement as a predictor of recurrence of AF after catheter ablation (Suksaranjit et al., 2015), recovery of LV ejection fraction after catheter ablation (Prabhu et al., 2017), and mortality (Neilan et al., 2013). In our study, LV delayed enhancement was found in 9% of AF patients, and was independently associated with reduced hyperemic MBF.

### 4.1 Limitations

There are some limitations in the present study. First, the use of vasoactive and antiarrhythmic medications was more common in patients with AF than in the control subjects. Second, as we did not perform rest dynamic CTP, it was not possible to analyze resting MBF and coronary flow reserve. Third, investigation of endothelial function using cold pressor test or acetylcholine before and after catheter ablation is an attractive approach to explore physiology of MBF in AF. However, these tests are not routinely performed as part of our clinical practice at our institution and could not be included in this retrospective study. Force, we could not measure the total durations of systole and diastole averaged per minute, to determine whether extravascular compression was different in AF patients compared to controls in SR and present what the effects of ablation were on the duration of systole and diastole because the ECG was monitored using the scanner's device for ECG gating. Therefore, it was difficult to detect component waves except for the R wave, and impossible to determine diastolic and systolic durations. Finally, performing CTP in addition to CCTA increases the ionizing radiation dose to the patient and also the iodinated contrast dose. Continued efforts need to be made to reduce radiation exposure and contrast agent dose.

## 5 CONCLUSION

Hyperemic MBF during pre‐ablation CT was lower in patients who were in AF than in the control group. The MBF impairment improved after restoration of SR by catheter ablation indicating that MBF abnormalities in AF patients are caused primarily by AF itself. CT evaluation of hyperemic MBF before and after catheter ablation may offer valuable insights into the pathogenesis of AF and the effects of catheter ablation. Further research is required to explore the potential clinical utility of hyperemic MBF in AF patients, in evaluation such as risk of cardiovascular event or recurrence of AF.

## CONFLICT OF INTEREST

Department of Advanced Diagnostic Imaging which Dr Kitagawa chairs is an endowment department supported by Siemens Healthcare K.K. and FUJIFILM Medical Co., Ltd. All the authors have nothing to disclose.

## AUTHOR CONTRIBUTION

Guarantor of integrity of entire study, Hajime Sakuma; study concepts/study design or data acquisition or data analysis/interpretation, all authors; literature research, Masafumi Takafuji, Kakuya Kitagawa, Satoshi Nakamura, and Hajime Sakuma; clinical studies, Masafumi Takafuji, Kakuya Kitagawa Takanori Kokawa, Yoshihiko Kagawa, Satoshi Fujita, Tomoyuki Fukuma, and Eitaro Fujii; statistical analysis, Masafumi Takafuji and Kakuya Kitagawa; and manuscript editing, Masafumi Takafuji, Kakuya Kitagawa, Satoshi Nakamura, and Hajime Sakuma.

## DISCLOSURE STATEMENT

Department of Advanced Diagnostic Imaging which Dr Kitagawa chairs is an endowment department supported by Siemens Healthineers Japan. All other authors have nothing to disclose.
